# Supplementary material for: miR-34b/c-5p/CXCL10 Axis Induced by RSV Infection Mediates a Mechanism of Airway Hyperresponsive Diseases
Source: Biology (Basel). 2023 Feb 16;12(2):317. doi: 10.3390/biology12020317 (PMC9953223; doi:10.3390/biology12020317)
Supplement: Supplementary file 1 [file biology-12-00317-s001.zip › biology-2183256-supplementary.pdf]

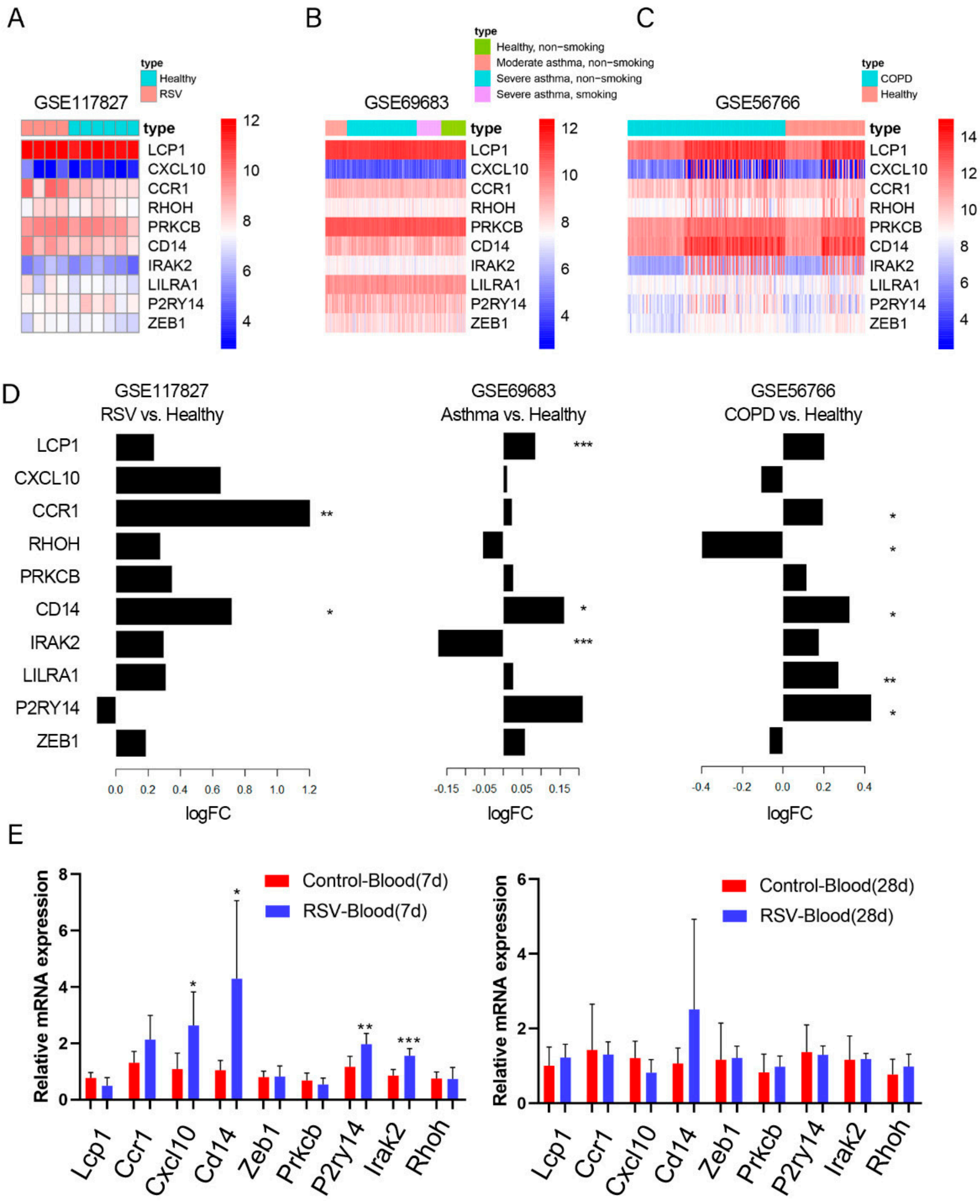

Figure S1. A synergistic analysis of Hub genes in the blood of three diseases. A-C, Heatmaps of the expression profiles of 10 hub genes in the blood of RSV infection, asthma, and COPD. Each column showed a single sample, each row represented a marker gene, and its expression value is normalized among 50 genes. D, the barplot display of the differential analysis of the expression of 10 hub genes in the lung of RSV infection, asthma, and COPD (*$p < 0.05$, ** $p < 0.01$, and*** $p < 0.001$ vs. Healthy). E, detection of mRNA levels of hub genes in blood of RSV-induced model using qRT-PCR (n = 6) (*$p < 0.05$, ** $p < 0.01$ and*** $p < 0.001$ vs. Control).

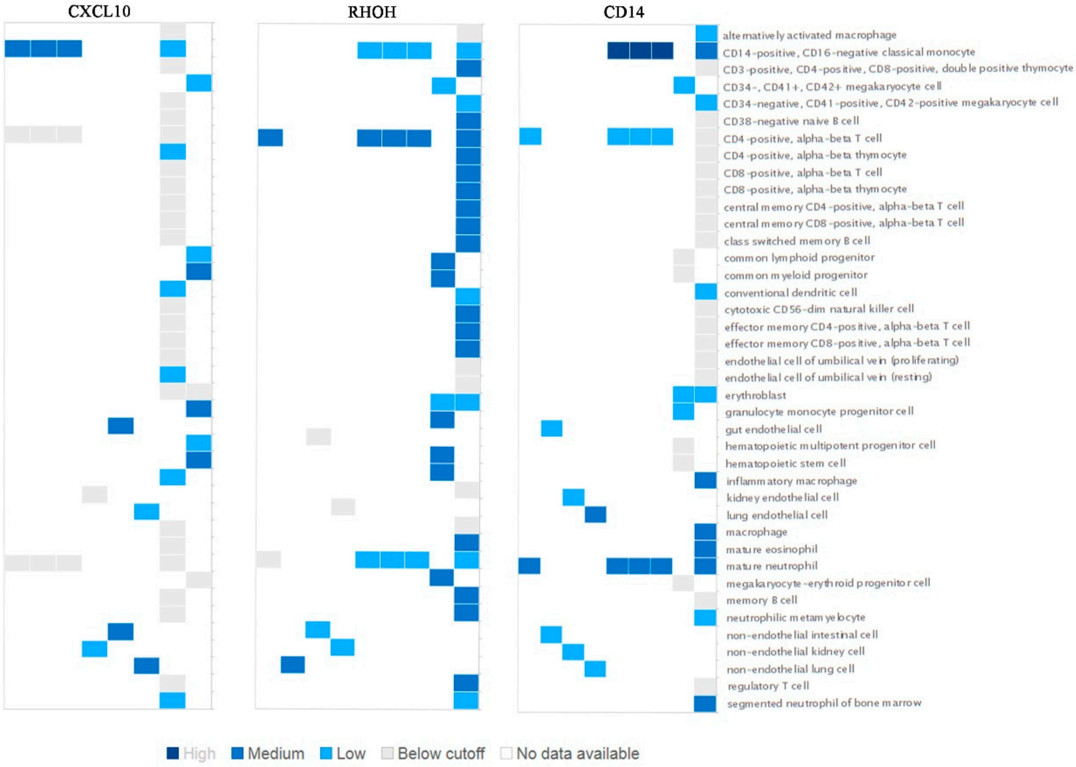

Figure S2. Expression distribution of CXCL10, RHOH, and CD14 in cell types. Using the Expression Atlas database (http://www.ebi.ac.uk/gxa/home) to analyze the expression distribution of hub genes CXCL10, RHOH, and CD14 in cell lines.

Table S1. Primers for the quantitative RT-PCR.

Genes	Sequences
LCP1-F (Mouse)	TCTGTGCCAGACACGATTGACG
LCP1-R (Mouse	GAGGCAGAGTTCAGAGCCAAGT
CCR1-F (Mouse)	GCCAAAAGACTGCTGTAAGAGCC
CCR1-R (Mouse)	GCTTTGAAGCCTCCTATGCTGC
CXCL10-F (Mouse)	ATCATCCCTGCGAGCCTATCCT
CXCL10-R(Mouse)	GACCTTTTTTGGCTAAACGCTTTC
CD14-F (Mouse)	TTGAACCTCCGCAACGTGTCGT
CD14-R (Mouse)	CGCAGGAAAAGTTGAGCGAGTG
ZEB1-F (Mouse)	ATTCAGCTACTGTGAGCCCTGC
ZEB1-R (Mouse)	CATTCTGGTCCTCCACAGTGGA
PRKCB-F (Mouse)	CCAAGATGACGATGTGGAGTGC
PRKCB-R (Mouse)	CTCCATCACAAAGTACAGGCGG
PZRY14-F (Mouse)	ACCTCCGTCAAGAGGAAGTCCA
PZRY14-R (Mouse)	GCTGTAGTGACCTTCCGTCTGA
IRAK2-F (Mouse)	GGACAGATTCCTACAGGCAGAG
IRAK2-R (Mouse)	GGTAGATGAGGCTGTGGAACTG
RHOH-F (Mouse)	TGGATGGCATCCAGATCAGCCT
RHOH-R (Mouse)	GTAGCACATCAGTACCACGTCTG
GAPDH-F (Mouse)	CATCACTGCCACCCAGAAGACTG
GAPDH-R (Mouse)	ATGCCAGTGAGCTTCCCGTTCAG
CXCL10-F (Human)	GGTGAGAAGAGATGTCTGAATCC
CXCL10-R (Human)	GTCCATCCTTGGAAGCACTGCA
CD14-F (Human)	CTGGAACAGGTGCCTAAAGGAC
CD14-R (Human)	GTCCAGTGTCAGGTTATCCACC
RHOH-F (Human)	TGGATGGCATCCAGATCAGCCT
RHOH-R (Human)	GCCACAGAGTAGCACATCAGCA
GAPDH-F (Human)	GTCTCCTCTGACTTCAACAGCG
GAPDH-R(Human)	ACCACCCTGTTGCTGTAGCCAA
miR-34b-5p-F	CGCGTAGGCAGTGTCATTAGC
miR-34b-5p-R	AGTGCAGGGTCCGAGGTATT
miR-34b-5p(RT Primer)	GTCGTATCCAGTGCAGGGTCCGAGGTATTCG-CACTGGATACGACCAATCA
miR-34c-5p-F	CGCGAGGCAGTGTAGTTAGCT
miR-34c-5p-R	AGTGCAGGGTCCGAGGTATT
miR-34c-5p (RT Primer)	GTCGTATCCAGTGCAGGGTCCGAGGTATTCG-CACTGGATACGACGCAATC
RSVF-F	CACCTTGTTGGAAACTGCACACATC
RSVF-R	CAGTACCATCCTCTGTCGGTTCTTG
U6-F	CTCGCTTCGGCAGCACA
U6-R	AACGCTTCACGAATTTGCGT

Table S2. Expression changes of miR34b/c-5p in the datasets.

DataSets	miRNA	logFC	*p*-Value
GSE62306	hsa-miR-34c-5p	−3.10729	0.000101
	hsa-miR-34b-5p	−2.33405	0.002036
GSE33336	hsa-miR-34c-5p	−1.79645	0.030761
	hsa-miR-34b-5p	−1.41812	0.025615
GSE142237	hsa-miR-34c-5p	−1.05808	0.011845
	hsa-miR-34b-5p	−1.89674	0.013616

Table S3. Ontological analysis of common DEGs.

GO	Term	*p*-value	Genes
Biological Process	cytokine-mediated signaling pathway (GO:0019221)	6.25×10^{-5}	CCR1;CXCL10;ZEB1;TNFSF14;IRAK2;LCP1
	cellular response to molecule of bacterial origin (GO:0071219)	1.19×10^{-4}	CXCL10;PDE4B;CD14
	T cell activation (GO:0042110)	1.37×10^{-4}	TNFSF14;RHOH;LCP1
	cellular response to lipopolysaccharide (GO:0071222)	1.51×10^{-4}	CXCL10;PDE4B;CD14
	positive regulation of monocyte chemotaxis (GO:0090026)	1.70×10^{-4}	CCR1;CXCL10
	positive regulation of mononuclear cell migration (GO:0071677)	2.14×10^{-4}	CCR1;CXCL10
	regulation of monocyte chemotaxis (GO:0090025)	2.88×10^{-4}	CCR1;CXCL10
	MyD88-dependent toll-like receptor signaling pathway (GO:0002755)	5.04×10^{-4}	IRAK2;CD14
	response to lipopolysaccharide (GO:0032496)	7.21×10^{-4}	CXCL10;PDE4B;CD14
	negative regulation of transport (GO:0051051)	9.61×10^{-4}	MCTP1;PRKCB
Molecular Function	histone threonine kinase activity (GO:0035184)	0.00802	PRKCB
	cAMP-dependent protein kinase regulator activity (GO:0008603)	0.00802	CXCL10
	G-protein coupled nucleotide receptor activity (GO:0001608)	0.00916	P2RY14
	C-C chemokine binding (GO:0019957)	0.00916	CCR1
	G-protein coupled purinergic nucleotide receptor activity (GO:0045028)	0.00916	P2RY14
	protein kinase C activity (GO:0004697)	0.01144	PRKCB
	long-chain fatty acid-CoA ligase activity (GO:0004467)	0.01144	ACSL1
	calcium-dependent protein serine/threonine kinase activity (GO:0009931)	0.01258	PRKCB
	metal ion binding (GO:0046872)	0.01367	MCTP1;PHACTR1;LCP1
	racemase and epimerase activity, acting on carbohydrates and derivatives (GO:0016857)	0.01372	PHACTR1
Cellular Component	recycling endosome (GO:0055037)	0.00819	MCTP1;RAB8B
	trans-Golgi network transport vesicle (GO:0030140)	0.01712	RAB8B
	anchored component of external side of plasma membrane (GO:0031362)	0.02051	CD14
	intrinsic component of external side of plasma membrane (GO:0031233)	0.02501	CD14
	lytic vacuole membrane (GO:0098852)	0.02911	AP1S2;LITAF
	lysosomal membrane (GO:0005765)	0.04367	AP1S2;LITAF
	contractile actin filament bundle (GO:0097517)	0.04503	LCP1
	stress fiber (GO:0001725)	0.04503	LCP1

peroxisomal membrane (GO:0005778)	0.04503	RAB8B
anchored component of plasma membrane (GO:0046658)	0.04503	CD14

Table S4. Pathway enrichment analysis of common DEGs.

**Term**	***p*-value**	**Genes**
NF-kappa B signaling pathway	1.72×10^{-4}	TNFSF14;PRKCB;CD14
Chemokine signaling pathway	0.001299	CCR1;CXCL10;PRKCB
Cytokine-cytokine receptor interaction	0.004478	CCR1;CXCL10;TNFSF14
MAPK signaling pathway	0.004521	PRKCB;CD14;CACNA1E
Morphine addiction	0.004868	PRKCB;PDE4B
Amoebiasis	0.005402	PRKCB;CD14
Toll-like receptor signaling pathway	0.00631	CXCL10;CD14
Parathyroid hormone synthesis, secretion and action	0.006547	PRKCB;PDE4B
Leukocyte transendothelial migration	0.007282	PRKCB;RHOH
Lysosome	0.008723	AP1S2;LITAF
